# Specialized outpatient care for maternal and child health in PlanificaSUS areas

**DOI:** 10.11606/s1518-8787.2023057005336

**Published:** 2024-04-01

**Authors:** Guilherme Barbosa Shimocomaqui, Eliana Tiemi Masuda, Victoria Gouveia de Souza, Ana Karina de Sousa Gadelha, Ilana Eshriqui

**Affiliations:** I Hospital Israelita Albert Einstein Centro de Estudos, Pesquisa e Prática em Atenção Primária à Saúde e Redes São Paulo SP Brasil Hospital Israelita Albert Einstein. Centro de Estudos, Pesquisa e Prática em Atenção Primária à Saúde e Redes. São Paulo, SP, Brasil; II Faculdade de Ciências da Saúde Albert Einstein Programa de Graduação em Enfermagem São Paulo SP Brasil Faculdade de Ciências da Saúde Albert Einstein. Programa de Graduação em Enfermagem. São Paulo, SP, Brasil

**Keywords:** Ambulatory Care, Health Services Accessibility, Maternal-Child Health Services, Delivery of Health Care

## Abstract

**OBJECTIVE:**

To describe the organization of specialized outpatient clinics, according to the Secondary Outpatient Care Unit (SOCU) model according to the health care planning (HCP) methodology.

**METHODS:**

This is a descriptive and cross-sectional study, which used secondary data from the PlanificaSUS project. It was carried out in 16 outpatient clinics specialized in maternal and child care, distributed in the five Brazilian geographic regions. A structured questionnaire was used for self-assessment on the implementation of 12 parameters in two moments, in 2019 and in 2020. These parameters are related to the care, educational, and supervisory functions set out in the SOCU model.

**RESULTS:**

In 2019, only 37.5% (six) of the outpatient clinics completed at least one parameter related to the care function, most frequently the multiprofessional team with interdisciplinary action (completed in 18.8% of the outpatient clinics). No parameters from the educational and supervisory functions were completed at this initial stage. In 2020, on the other hand, parameters related to the care function also showed higher frequency, higlighting the use of the same criterion by primary care teams and outpatient clinics for risk stratification (completed in 68.8% of the outpatient clinics). In the educational and supervisory functions, parameters related to the encounter between primary care teams and outpatient clinics for case management development, integrated training promotion, and close communication bond among these professionals also increased. Completion of these three parameters was identified in 25%, 25%, and 37.5% of the outpatient clinics, respectively.

**CONCLUSIONS:**

The planning methodology fostered reflection and discussion about the (re)organization of the work process and contributed to changes in maternal and child health care practices within specialized outpatient care, integrated with primary health care (PHC), from the perspective of care networks. We believe that such advances enhance access and equitable care for high-risk pregnant women and children in different geographical regions of Brazil.

## INTRODUCTION

The traditional health care systems present challenges for both developed and developing countries since they do not respond according to demographic and epidemiological situations. In developing countries like Brazil, the healthcare system must address the epidemiological situation of the triple burden of diseases, with a prevalence of chronic conditions, infectious diseases, and external causes.^1^. However, traditional, fragmented systems, organized in silos, predominantly address acute conditions and exacerbations of chronic conditions in a reactive and episodic manner^4,5^.

As a strategy to overcome the fragmentation of care and respond to the Country’s epidemiological situation, in 2010, Brazil published guidelines for organizing Health Care Networks within the Brazilian public health system (Brazilian Unified Health System – SUS)^3^. These guidelines propose strategies for care coordination through primary health care (PHC) and economies of scale to integrate services, enhance and qualify access, as well as to resolve health issues more effectively.^6,7^. Thus, the Health Care Network (HCN) is characterized by the formation of horizontal and integrated relationships among care centers, aiming to respond to the health needs of the population^3,5^. Since it presents greater systemic rationality, such a proposal also contributes to a better allocation of resources^8^. Considering the high rates of maternal and infant mortality in Brazil and worldwide^9^, the Stork Network (Rede Cegonha) stands out as one of the thematic networks prioritized by the SUS. Its aims to organize a care model for childbirth, delivery, and maternal and child health, ensuring proper reception and, above all, reducing avoidable deaths of children, pregnant women, and individuals in postpartum^7,10,11^.

Given this scenario, Health Care Planning (HCP) is a methodology proposed by the *Conselho nacional de Secretários de Saúde* (Conass – National Council of Health Secretaries) to organize the HCN. It is used as a management and organization tool for the Specialized Outpatient Care (SOC) in coordination with PHC^12^. Considering the scarcity of evidence regarding the practices of SOC, as well as the absence of national policies directed towards this point of attention, there is a cognitive gap for SOC within the SUS. This is not due to a lack of certain specialties, but rather due to the inefficiency of the fragmented organization, resulting in drawbacks in providing comprehensive, equitable, and appropriate care^6,13^. To overcome this problem, the HCP methodology adopts the Secondary Outpatient Care Unit (SOCU) model as a new form of organization between PHC and SOC^6,13^, based on the Chronic Conditions Care Model (CCCM), in line with clinical guidelines. According to the SOCU model, the Specialized Outpatient Care (SOC) provides a range of services comprising a multiprofessional team and health technologies to develop educational, supervisory/institutional support, and research functions, in addition to care duties (including telemedicine)^6^.

In this perspective, SOC keeps a horizontal and collaborative relationship with PHC, forming a single clinical microsystem^5,6^. Even in different services, individuals work together regularly, supporting users and families living in a specific territory to provide healthcare tailored to their needs^6,14^.

Aspiring to implement the HCP methodology on a large scale, the Sociedade Beneficente Israelita Brasileira Albert Einstein has been executing the PlanificaSUS project since 2018, using the Support Program for Institutional Development of the Unified Health System (Proadi-SUS). PlanificaSUS aims to support the technical staff of state health departments in implementing the planning methodology, to strengthen the role of PHC and SOC in organizing the HCN within the SUS. (https://planificasus.com.br/). Considering the challenge of improving maternal and child care and the gap in the literature regarding network organization, according to the SOCU model, this study aims to describe the organization of outpatient clinics for high-risk pregnant women and children participating in PlanificaSUS. This description is based on the care, educational, and supervisory functions of the SOCU model.

## METHODS

### Design and Setting

This is a cross-sectional descriptive study carried out between 2019 and 2020, using secondary data from the PlanificaSUS project. This study was approved by the Research Ethics Committee of Hospital Israelita Albert Einstein (opinion no. CEP 3.674.106, CAAE 12395919.0.0000.0071).

The project is implemented in 24 health regions across 18 participating federal units, encompassing one outpatient clinic per region. These clinics were selected by state health departments to serve as showcase for organization based on the SOCU model in their respective health regions. In these settings, priority care pathways were adopted (hypertension and diabetes, health of the elderly, or maternal and child health) based on epidemiological criteria. For this study, 18 outpatient clinics were eligible to participate. They are located in regions that prioritized the maternal and child health care pathway.

Two outpatient clinics were excluded because they abstained from participating in the interest parameters self-assessment. Thus, we conducted the research with a sample of 16 outpatient clinics located in the five geographical regions of Brazil (four in the North, five in the Northeast, three in the Midwest, two in the South, and two in the Southeast).

### Data Collection

Secondary data were used, collected in two stages (2019 and 2020), during the PlanificaSUS project operationalization. In this period, themes such as network diagnosis, territory, population-based management, access to HCN, and care management were developed through a tutoring process. The activities conducted included moments of theoretical-conceptual alignment, reflection, and changes in work processes. These activities were facilitated by local actors (tutors), with the participation of healthcare professionals from the health units, while also involving the support of external actors (tutor analysts) under the management of the PlanificaSUS team.

The operationalization of activities in health units began in July 2019, when the outpatient clinic tutors were instructed to respond, with the support of the respective service professionals, to a structured questionnaire to diagnose compliance parameters with the SOCU model (first stage of data collection). The second stage of collection took place in the second half of 2020, using the same questionnaire to assess possible changes after the development of the aforementioned themes during the PlanificaSUS execution.

In both stages, the responses to 12 parameters related to the care, educational, and supervisory/institutional support functions outlined in the SOCU model were considered (Table). The research function was not considered as it was not developed during this period of PlanificaSUS execution. The responses were self-reported by the actors mentioned earlier. The possible responses for each parameter of the questionnaire were: “Does not exist,” “Partially,” and “Completed.”

**Table:** Percentage of outpatient clinics (n = 16) reporting parameter completion related to the functions outlined in the SOCU model, Brazil, in 2019 and in 2020.

Parameters	Stage 1 (2019)	Stage 2 (2020)	Difference between the two stages of self-assessment
		
	n (%)	n (%)	Percentage points
Care function			
1. The PHC and SOC teams use the same criteria for user risk stratification	1 (6.2)	11 (68.8)	62.6
2. Access is regulated by the teams of the proven PHC	1 (6.2)	7 (43.8)	37.6
3. The flow and scheduling criteria of the shared users was defined and agreed upon by the PHC and SOC teams, and approved in the CIR	1 (6.2)	5 (31.2)	25
4. The team is multiprofessional, with proven interdisciplinary performance	3 (18.8)	14 (87.5)	68.7
5. Continuous care is fully offered to all users in first care and subsequent care is organized in partial cycles, according to the care plan	0 (0)	3 (18.8)	18.8
Educational function			
6. The service holds events aimed at educating users, in partnership with PHC teams in their territory	0 (0)	1 (6.2)	6.2
7. Meetings are held between the PHC teams and the SOC to prepare a care plan	0 (0)	3 (18.8)	18.8
8. Meetings are held between PHC and PHC teams to develop case management	0 (0)	4 (25.0)	25
9. The service promotes integrated training for PHC and SOC professionals	0 (0)	4 (25.0)	25
10. Technical-scientific meetings are organized among professionals to study clinical guidelines, technical reference documents, and their updates	0 (0)	3 (18.8)	18.8
Supervisory function/institutional support			
11. A close communication link is maintained between SOC and PHC professionals, via telephone, e-mail, and social networks	0 (0)	6 (37.5)	37.5
12. The SOC team carries out supervisory actions (institutional support) for municipalities, aiming to support PHC teams in improving processes related to prioritized lines of care	0 (0)	4 (25.0)	25

SOC: Specialized Outpatient Care; PHC: Primary Health Care; CIR: Comissão Intergestores Bipartite.

The questionnaire used in both stages was made available on the project’s execution monitoring platform (e-Planifica – https://planificasus.com.br). After the self-assessment and filling out the Excel instrument, the file was uploaded to the e-Planifica system by each unit’s tutor. For this study, we used the consolidated uploaded files from the 16 participating outpatient clinics.

### Data Analysis

To facilitate response interpretation, the 12 interest parameters were considered in a dichotomous manner (“Does not exist” or “Completed”). Hence, responses categorized as “Partially” were recategorized as “Does not exist.” The variables of interest in this study pertain to the overall completion percentage of the evaluated parameters, according to the three functions outlined in SOCU and the location within the Brazilian geographical regions.

The implementation percentages of the parameters were calculated based on the number of outpatient clinics reporting the parameter as implemented (“Completed”) divided by the total number of outpatient clinics in the study for each evaluation stage (first in 2019 and second in 2020). The function implementation percentage by geographical region (North, Northeast, Midwest, Southeast, and South) (Figure) was calculated using the ratio of the score sums obtained for each function by geographical region divided by the maximum score (considering the number of parameters for each function multiplied by the number of participating outpatient clinics in each of the five Brazilian regions).

**Figure:**
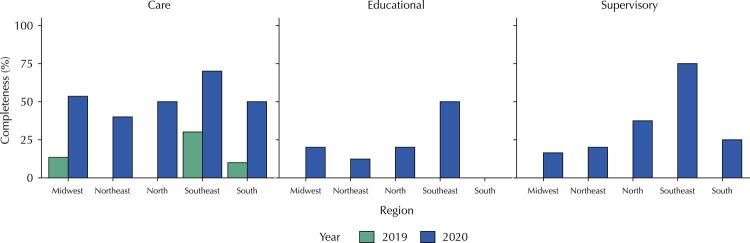
Compliance parameters with the SOCU model, referring to the functions of specialized Outpatient Care by region, in Brazil, in 2019 and 2020.

SOCU: Point of Secondary Outpatient Care.

Microsoft Excel and R (v.4.1.0) software were used to perform the statistical analysis and plot the bar chart.

## RESULTS

Sixteen outpatient clinics completed both self-assessment stages on the implementation of the SOCU model. From the first diagnosis conducted in 2019 using the structured questionnaire, 62.5% (n = 10) of the outpatient clinics lacked completed parameters. Among the six clinics that reported having some completed parameters, two were located in the South, two in the Midwest, and two in the Southeast. At that time, no parameters related to the educational and supervisory functions were reported as completed, while the most frequently completed parameter was the multiprofessional team with interdisciplinary action (related to care function), completed in 18% of the outpatient clinics.

The Table presents the consolidated diagnosis of parameters in both evaluation stages. In the second self-assessment conducted in 2020, 93.7% (n = 15) of the outpatient clinics reported having at least one “Completed” parameter. The parameter with the highest percentage increase was the multiprofessional team, followed by the usage of the same criteria by PHC and SOC teams for user risk stratification, both related to the care function. Still within the care function, continuous attention was the parameter with the lowest percentage of completion in the second assessment. Notably, this was the only care parameter that was not implemented in any of the outpatient clinics in the first assessment stage.

We observed that the parameter with the smallest difference in completion between the stages of this study was the organization of events aimed at educating users in partnership with PHC teams in the territory, related to the educational function. The parameters with the highest percentage of completion in 2020 among those related to the educational function were the meetings between PHC and SOC teams for case management development, and the promotion of integrated training for PHC and SOC professionals.

Within the supervisory function, the parameter related to close communication links between PHC and SOC professionals increased notably in reported completion.

The Figure depicts the difference in the sum of completed parameters for each of the three PHC functions studied in both self-assessment stages, according to the units’ location in the Brazilian geographical regions.

## DISCUSSION

This study described parameters related to three functions of the SOCU model in outpatient clinics distributed across the five geographical regions of Brazil. It was evident that before the implementation of the HCP methodology with the PlanificaSUS project, the care function seemed to be partially implemented in outpatient clinics in the South, Southeast, and Midwest. Nonetheless, the educational and supervisory functions were not developed in any of the studied clinics. In the second assessment, we identified a significant progress in all three functions outlined in the SOCU model, especially in the care function. We believe that these advances contributed significantly to the work process organization that enhances access and care for high-risk pregnant women and children in different geographical regions of Brazil.

In the first assessment stage of the study, only parameters related to the care function appeared as already implemented processes, which corroborates the premise that the existing silo model works only with this function^6^. However, despite being partially implemented, we observed that only the minority of outpatient clinics complied to the function’s parameters.

The care parameter that developed the most between the two self-assessment stages is the one related to multiprofessional team with interprofessional action. Peduzzi^15^ emphasizes that “multiprofessional teamwork consists of collective work that is configured in the reciprocal relationship between multiple technical interventions and the interaction between agents from different professional areas” (our translation). In this sense, to avoid the “cluster team” configuration, it is essential to communicate and coordinate the actions among the actors with an integrating work perspective. This involves considering a common care project, flexibility in task division, and interdependent technical autonomy based on differences in specialties^16^. Developing teamwork skills, reflecting on, and critically discussing the professionals’ performance, as well as defining professional limits and autonomy, are essential steps to break free from the fragmented health training model and practices, aiming at strengthening multiprofessional and interdisciplinary work to address the population’s health needs^17^.

We highlight that the SOCU model supports and facilitates these aspects since it provides care activities with continuous attention, based on clinical guidelines from multiprofessional teams with interprofessional action^6,13^. Such characteristics represent a remarkable difference between the SOCU model and the silo model. From the perspective of the continuous care cycle, there is a recommendation for integrated assessment and the collective construction of the care plan, stemming from sequential individual appointments, in a previously established order^18^. This study identified that none of the outpatient clinics offered continuous care in the first assessment stage, while in the second stage, after about a year implementing the HCP methodology with PlanificaSUS, around 20% had already started engaging in care activities with continuous attention. It is important to emphasize that the outpatient clinics participating in PlanificaSUS began to plan continuous care implementation processes during the first quarter of 2020. However, this occasion coincided with the onset of the COVID-19 pandemic in Brazil. This event required an adaptation to a virtual mode and a rescheduling to prioritize addressing COVID-19 demands. In this sense, such progress is considered relevant, although it is the parameter of the care function that developed the least in the study period.

We also observed advancements in other parameters related to the care function, which involve the integration between primary care and outpatient clinic teams, since they address issues related to the alignment of clinical guidelines between services, access regulation, flows, and care sharing. The outpatient clinics improved integration with PHC with the qualification of work processes using the HCP methodology. Especially concerning maternal and child health, it is worth noting that the Stork Network (Rede Cegonha) advocates for timely access for high-risk pregnant women to specialized outpatient clinics^7,10^. For this purpose, we need a network integration and good communication among healthcare. From this point of view, we must consider building bonds between users and professionals, as well as among professionals from different services, so care sharing between primary care services and specialized outpatient care can be effective. This involves recognizing the role of PHC as care coordinator^6,7,13,18^.

Regarding the educational function of the SOCU model, this study highlighted a gap in the organization of events aimed at user education performed by outpatient clinics in partnership with PHC. Nevertheless, it is important to emphasize that the SOCU model recommends developing educational actions during continuous care cycles, aimed at users, to strengthen comprehensiveness, health education, self-care, and supported self-care^6,13,18,21,22^.

Regarding the educational function of outpatient clinics with PHC teams, the results of this study highlight the synergy of the SOCU model with permanent health education^6,13,17,18,23^. Yet little expressive, there was an increase in the implementation of joint activities with PHC, indicating the strengthening of matrix support developed between teams. In this model, outpatient clinic professionals ensure technical-pedagogical and technical-assistance support to reference professionals^6,13,18,23^. Such strategies value meaningful learning at work through exchanges between outpatient clinic specialists and PHC reference teams, with great potential to transform practices in service routine^24,25^.

The study highlighted small advances concerning the close communication link between outpatient clinic and PHC professionals, as well as the supervisory actions. The goal was to support these teams in improving processes related to maternal and child care. It is noteworthy that supervision/institutional support contributes to shared and participatory management among services, managers, professionals, and users. This support promotes institutional development since it presupposes mediation between technical knowledge and ethical-political commitment, considering that every management is a product of relationships between people^26^. This framework corroborates the praxis of the outpatient clinics’ supervisory function organized by the SOCU model, which seeks to break with traditional and bureaucratized logics of communication between different points of the HCN.

Several factors might be related to the changes highlighted by this study in the work processes of specialized services according to the SOCU model. A systematic review exposes facilitators and barriers to implement innovations in secondary care^29^. The lack of human, material, and financial resources, as well as the integration of workflow, and organizational readiness stood out among the main barriers. On the other hand, an open and supportive organizational culture, training, education, and knowledge, as well as recognizing added value to users, facilitated the implementation of innovations in specialized services^29^.

In this context, the HCP methodology provides elements to mitigate some of these barriers and facilitate the implementation of outpatient services in the SOCU model. Establishing clinical guidelines, flows, and protocols in the care network, moments of theoretical and conceptual alignment, as well as reflection, discussion, and agreements on work practices by tutoring stood out among these elements.

Even though challenges in accessing specialized care persist, there were advances to recognize the role of PHC as the HCN coordinator^30,31^. Notably, the recent publication of the *Política Nacional de Atenção Especializada em Saúde* (National Policy of Specialized Healthcare)^32^, which advocates guidelines, dimensions, and structuring axes aimed at expanding access, person-centered care, including greater integration among professional practices, promoting continuing education, and strengthening PHC as a resolving, ordering, and coordinating care entity.

Among the limitations of this study, we highlight the potential information bias since the data relied on secondary information from self-assessment by representatives of health services. Among the strategies implemented to mitigate the possibility of divergence between what professionals reported and the actual situation in the outpatient clinics, it is worth mentioning that we used a standardized instrument and the support of external actors during the self-assessment activities by the services outlined in PlanificaSUS. Among the strengths, we highlight that this study was the first to describe the organization of outpatient clinics as recommended by the SOCU model, based on the HCP methodology, including units from all Brazilian geographic regions.

In addition, we highlight elements that connect with the structuring and organization of specialized care in the SOCU model, from the HCN perspective. Particularly, we must highlight the relevance of the HCP methodology when promoting reflections and inducting practices aimed at (re)organizing the work process of health teams in training for access and care of high-risk pregnant women and children in the HCN.

We observe the need for future implementation research to identify facilitators and barriers to organize outpatient clinics in an integrated manner with PHC in different Brazilian contexts. In particular, studies that encompass the organization of specialized care in the SOCU model are necessary not only to investigate the determinants of implementation but also its outcomes. These outcomes relate to changes and sustainability of practices among healthcare professionals and managers in a multiprofessional, collaborative, guideline-based, comprehensive, and equitable manner, as well as the impact on the care of high-risk pregnant women and children.

## CONCLUSION

This study allowed us to verify the progress in implementing the care, educational, and supervisory functions according to the SOCU model in outpatient clinics that prioritized the maternal and child care line in PlanificaSUS. Furthermore, it highlights the predominance of the fragmented silo model in the Brazilian scenario by finding only parameters related to the care function, albeit incipient, in the first evaluation stage. The HCP methodology contributed to the (re)organization of the work processes of healthcare teams and induced changes in maternal and child health care practices in outpatient clinics, integrated with PHC, according to the SOCU model. From this research, the need for further studies aimed at elucidating the impact of the organization of integrated outpatient clinics in a network with PHC on health outcomes in the Brazilian population unfolds.
