# Supplementary material for: Specialized outpatient care for maternal and child health in PlanificaSUS areas
Source: Rev Saude Publica. 2024 Apr 1;57(Suppl 3):3s. doi: 10.11606/s1518-8787.2023057005336 (PMC11037908; doi:10.11606/s1518-8787.2023057005336)
Supplement: Translation

# Translation: Atenção ambulatorial especializada à saúde materno-infantil em regiões do PlanificaSUS

**Authors:** Guilherme Barbosa Shimocomaqui, Eliana Tiemi Masuda, Victoria Gouveia de Souza, Ana Karina de Sousa Gadelha, Ilana Eshriqui

**Affiliations:** I Brasil Hospital Israelita Albert Einstein. Centro de Estudos, Pesquisa e Prática em Atenção Primária à Saúde e Redes. São Paulo, SP, Brasil; II Brasil Faculdade de Ciências da Saúde Albert Einstein. Programa de Graduação em Enfermagem. São Paulo, SP, Brasil

**Keywords:** Assistência Ambulatorial, Acesso aos Serviços de Saúde, Serviços de Saúde Materno-Infantil, Atenção à Saúde

## Abstract

**OBJETIVO:**

Descrever a organização de ambulatórios especializados, conforme o modelo Ponto de Atenção Secundária Ambulatorial (Pasa), por meio da metodologia da planificação de atenção à saúde (PAS).

**MÉTODOS:**

Trata-se de um estudo descritivo e transversal, que utilizou dados secundários do projeto PlanificaSUS. Foi realizado em 16 ambulatórios especializados na linha de cuidado materno infantil, distribuídos nas cinco regiões geográficas brasileiras. Um questionário estruturado foi utilizado para autoavaliação sobre a implantação de 12 parâmetros em dois momentos, em 2019 e em 2020. Esses parâmetros são relacionados às funções assistencial, educacional e de supervisão previstas no modelo Pasa.

**RESULTADOS:**

Em 2019, apenas 37,5% (seis) dos ambulatórios apresentaram, pelo menos, um parâmetro concluído, sendo com maior frequência o de equipe multiprofissional com atuação interdisciplinar (concluído em 18,8% dos ambulatórios), relacionado à função assistencial. Nenhum parâmetro das funções educacional e de supervisão estava concluído nesse primeiro momento. Já em 2020, os parâmetros relacionados à função assistencial também apresentaram maior frequência, destacando-se a utilização do mesmo critério pelas equipes da atenção primária e dos ambulatórios para estratificação de risco (concluídos em 68,8% dos ambulatórios). Nas funções educacional e supervisional, os parâmetros de encontro entre as equipes da atenção primária e dos ambulatórios para o desenvolvimento da gestão de caso, promoção de capacitação integrada e vínculo estreito de comunicação entre esses profissionais também aumentaram, identificando-se conclusão destes três parâmetros em 25%, 25% e 37,5% dos ambulatórios, respectivamente.

**CONCLUSÕES:**

A metodologia da planificação proporcionou reflexão e discussão acerca da (re)reorganização do processo de trabalho e contribuiu para a mudanças de práticas de cuidado à saúde materno-infantil na atenção ambulatorial especializada, de forma integrada com a atenção primária à saúde (APS), na perspectiva das redes de atenção. Acredita-se que tais avanços potencializam o acesso e cuidado equitativo de gestantes e crianças de alto risco nas diferentes regiões geográficas brasileiras.

## INTRODUÇÃO

Os sistemas de atenção à saúde tradicionais apresentam desafios tanto para os países desenvolvidos quanto para os que estão em desenvolvimento, por não responderem de acordo com as situações demográficas e epidemiológicas. Nos países em desenvolvimento, como o Brasil, o sistema de saúde deve atender a situação epidemiológica da tripla carga de doenças, com predomínio das condições crônicas, doenças infecciosas e causas externas^1^. No entanto, os sistemas tradicionais, fragmentados em silos, atendem predominantemente às condições agudas e aos eventos de agudizações das condições crônicas, de forma reativa e episódica^4,5^.

Como estratégia para superar a fragmentação da atenção e responder à situação epidemiológica do país, em 2010, o Brasil publicou uma diretriz de organização em Redes de Atenção à Saúde no âmbito do sistema público de saúde brasileiro (Sistema Único de Saúde – SUS)^3^, a qual propõe estratégias de coordenação do cuidado pela atenção primária à saúde (APS) e economia de escala, a fim de integrar os serviços, aumentar e qualificar o acesso, assim como a resolutividade dos problemas de saúde^6,7^. Assim, a Rede de Atenção à Saúde (RAS) se caracteriza pela formação de relações horizontais e integradas entre os pontos de atenção, de forma a responder às necessidades de saúde da população^3,5^. Uma vez que apresenta maior racionalidade sistêmica, tal proposta contribui ainda para melhor alocação dos recursos^8^. Considerando as altas taxas de mortalidade materno-infantil no Brasil e no mundo^9^, a Rede Cegonha destaca-se como uma das redes temáticas priorizadas pelo SUS, que tem como objetivo organizar um modelo de atenção ao parto, nascimento e atenção à saúde materno-infantil, assegurando um bom acolhimento e, sobretudo, a diminuição das mortes evitáveis de crianças, gestantes e pessoas em puerpério^7,10,11^.

Diante desse cenário, a Planificação da Atenção à Saúde (PAS) trata-se de uma metodologia proposta pelo Conselho Nacional de Secretários de Saúde (Conass) para a organização da RAS, utilizada como um instrumento de gestão e organização da Atenção Ambulatorial Especializada (AAE) em rede com a APS^12^. Cabe destacar que, considerando a escassez de evidências sobre as práticas de atuação da AAE, assim como a ausência de políticas nacionais direcionadas a este ponto de atenção, configura-se um vazio cognitivo da AAE no SUS, não pela falta de oferta de algumas especialidades, mas pela ineficiência da organização fragmentada, resultando em prejuízos na oferta de cuidado integral, equitativo e oportuno^6,13^. Para superação desta problemática, a metodologia da PAS adota o modelo do Ponto de Atenção Secundária Ambulatorial (Pasa) como uma nova forma de organização entre a APS e a AAE^6,13^, baseado no Modelo de Atenção às Condições Crônicas (Macc), em consonância com diretrizes clínicas. De acordo com o modelo Pasa, a AAE prevê uma carteira de serviços composta por equipe multiprofissional e tecnologias em saúde para o desenvolvimento das funções educacional, de supervisão/apoio institucional e de pesquisa, para além da assistencial (incluindo a teleassistência)^6^.

Nesta perspectiva, a AAE mantém uma relação horizontal e colaborativa com a APS, compondo um único microssistema clínico^5,6^. Mesmo em serviços diferentes, as pessoas trabalham juntas e de forma regular, atendendo usuários e famílias que vivem em um determinado território para desenvolver cuidados em saúde conforme suas necessidades^6,14^.

Com vistas à implantação da metodologia da PAS em larga escala, a Sociedade Beneficente Israelita Brasileira Albert Einstein executa, desde 2018, o projeto PlanificaSUS, via Programa de Apoio ao Desenvolvimento Institucional do SUS (Proadi-SUS). O PlanificaSUS tem como objetivo apoiar o corpo técnico das secretarias estaduais de saúde na implantação da metodologia da planificação, a fim de fortalecer o papel da APS e da AAE na organização da RAS no SUS (https://planificasus.com.br/). Considerando o desafio de qualificação do cuidado materno-infantil e a lacuna da literatura acerca da organização da rede, conforme o modelo Pasa, este estudo tem como objetivo descrever a organização de ambulatórios de gestantes e crianças de alto risco participantes do PlanificaSUS, a partir das funções assistencial, educacional e de supervisão do modelo Pasa.

## MÉTODOS

### Desenho e Cenário

Trata-se de um estudo descritivo transversal realizado entre 2019 e 2020, a partir de dados secundários do projeto PlanificaSUS. O presente estudo foi aprovado pelo Comitê de Ética em Pesquisa do Hospital Israelita Albert Einstein (parecer CEP 3.674.106, CAAE 12395919.0.0000.0071).

O projeto é executado em 24 regiões de saúde de 18 unidades federativas participantes, compreendendo um ambulatório por região, os quais foram elencados pelas secretarias estaduais de saúde para atuar como vitrine de organização a partir do modelo PASA em suas respectivas regiões de saúde. Nesses cenários, foram adotadas linhas de cuidado prioritárias (hipertensão e diabetes, saúde do idoso ou materno-infantil), elegidas a partir de critérios epidemiológicos. Para este estudo, foi elegível a participação de 18 ambulatórios, localizados em regiões que adotaram a linha de cuidado materno-infantil como prioritária.

Foram excluídos dois ambulatórios, por não participarem da autoavaliação dos parâmetros de interesse. Desta forma, a pesquisa foi conduzida com amostra de 16 ambulatórios localizados nas cinco regiões geográficas brasileiras (quatro no Norte, cinco no Nordeste, três no Centro-Oeste, dois no Sul e dois no Sudeste).

### Coleta de Dados

Foram utilizados dados secundários, coletados em duas etapas (2019 e 2020), no contexto de operacionalização do projeto PlanificaSUS, durante o qual as temáticas de diagnóstico da rede, território e gestão de base populacional, acesso à RAS e gestão do cuidado foram desenvolvidas por meio de processo de tutoria. As atividades desenvolvidas compreendiam momentos de alinhamento teórico-conceitual, reflexão e mudanças dos processos de trabalhos, os quais eram facilitados por atores locais (tutores), com a participação dos profissionais das unidades de saúde, contando ainda com o apoio de atores externos (analistas de tutoria) sob gestão da equipe do PlanificaSUS.

O início da operacionalização das atividades nas unidades de saúde se deu a partir de julho de 2019, quando os tutores dos ambulatórios foram orientados a responder, com o apoio dos profissionais dos respectivos serviços, um questionário estruturado, a fim de diagnosticar parâmetros de conformidade com o modelo Pasa (1ª etapa da coleta de dados). A segunda etapa da coleta foi realizada no segundo semestre de 2020, quando o mesmo questionário foi utilizado para verificar possíveis mudanças após o desenvolvimento das temáticas mencionadas acima, no contexto da execução do PlanificaSUS.

Em ambas as etapas, foram consideradas as respostas de 12 parâmetros referentes às funções assistencial, educacional e supervisão/apoio institucional previstas no modelo Pasa (Tabela). A função de pesquisa não foi considerada, por não ter sido desenvolvida neste período de execução do PlanificaSUS. As respostas foram autorreferidas pelos atores destacados anteriormente. As possíveis respostas para cada parâmetro do questionário eram: “Não existe”, “Parcialmente” e “Concluído”.

**Tabela:** Porcentagem de ambulatórios (n = 16) que relataram conclusão de parâmetros relacionados às funções previstas no modelo Pasa, Brasil, em 2019 e em 2020.

Parâmetros	Etapa 1 (2019)	Etapa 2 (2020)	Diferença entre as duas etapas de autoavaliação
		
	n (%)	n (%)	Pontos percentuais
Função assistencial			
1. As equipes da APS e AAE utilizam os mesmos critérios para estratificação de risco dos usuários	1 (6,2)	11 (68,8)	62,6
2. O acesso é regulado pelas equipes da APS comprovada	1 (6,2)	7 (43,8)	37,6
3. O fluxo e critérios de agendamento dos usuários compartilhados foi definido e pactuado pelas equipes da APS e AAE, e aprovado na CIR	1 (6,2)	5 (31,2)	25
4. A equipe é multiprofissional, com atuação interdisciplinar comprovada	3 (18,8)	14 (87,5)	68,7
5. A atenção contínua é oferecida integralmente para todos os usuários em primeiro atendimento e os atendimentos subsequentes são organizados em ciclos parciais, de acordo com o plano de cuidados	0 (0)	3 (18,8)	18,8
Função educacional			
6. O serviço organiza eventos voltados à educação de usuários, em parceria com as equipes de APS do seu território	0 (0)	1 (6,2)	6,2
7. São realizadas reuniões entre as equipes da APS com a AAE para elaboração de plano de cuidados	0 (0)	3 (18,8)	18,8
8. São realizados encontros entre as equipes da APS e AAE para desenvolvimento da gestão de caso	0 (0)	4 (25,0)	25
9. O serviço promove capacitação integrada para profissionais da APS e AAE	0 (0)	4 (25,0)	25
10. São organizadas reuniões técnico-científicas entre os profissionais para estudo das diretrizes clínicas, documentos técnicos de referência e suas atualizações	0 (0)	3 (18,8)	18,8
Função de supervisão/apoio institucional			
11. É mantido vínculo estreito de comunicação entre profissionais da AAE e da APS, via telefone, e-mail e redes sociais	0 (0)	6 (37,5)	37,5
12. A equipe AAE realiza ações de supervisão (apoio institucional) aos municípios, com o objetivo de apoiar as equipes APS na melhoria dos processos relativos às linhas de cuidado priorizadas	0 (0)	4 (25,0)	25

AAE: Atenção Ambulatorial Especializada; APS: atenção primária à saúde; CIR: Comissão Intergestores Bipartite.

O questionário utilizado em ambas as etapas foi disponibilizado na plataforma de monitoramento da execução do projeto (e-Planifica – https://planificasus.com.br). Após a autoavaliação e preenchimento do instrumento em Excel, o arquivo foi anexado no e-Planifica pelo tutor de cada unidade. Para este estudo, utilizou-se o consolidado dos arquivos anexados referentes aos 16 ambulatórios participantes.

### Análise de Dados

Objetivando facilitar a interpretação das respostas, os 12 parâmetros de interesse foram considerados de forma dicotômica (“Não existe” ou “Concluído”), de modo que as respostas “Parcialmente” foram recategorizadas como “Não existe”. As variáveis de interesse deste estudo referem-se à porcentagem geral de conclusão dos parâmetros avaliados, de acordo com três funções previstas no PASA e com a localização nas regiões geográficas brasileiras.

As porcentagens de implantação dos parâmetros foram calculadas a partir do número de ambulatórios que relataram o parâmetro implantado (“Concluído”) sobre o número total de ambulatórios do estudo por etapa de avaliação (primeira em 2019 e segunda em 2020). O cálculo da porcentagem de implantação das funções por região geográfica (Norte, Nordeste, Centro-Oeste, Sudeste e Sul) (Figura) foi realizado a partir da relação da soma da pontuação obtida para cada função por região geográfica, sobre a pontuação máxima (considerando o número de parâmetros para cada função multiplicado pelo número de ambulatórios participantes em cada uma das cinco regiões brasileiras).

**Figura:** Parâmetros de conformidade com o modelo Pasa, referentes às funções da Atenção Ambulatorial Especializada por região, no Brasil, nos anos de 2019 e 2020.

Pasa: Ponto de Atenção Secundária Ambulatorial.

Foram utilizados os softwares Microsoft Excel e Programa Estatístico R (v.4.1.0) para a realização das análises estatísticas e confecção do gráfico de barras.

## RESULTADOS

As duas etapas de autoavaliação sobre a implantação do modelo Pasa foram completadas por 16 ambulatórios. A partir do primeiro diagnóstico realizado em 2019 por meio do questionário estruturado, 62,5% (n = 10) dos ambulatórios não apresentavam nenhum parâmetro concluído. Entre os seis que referiram ter algum parâmetro concluído, dois localizavam-se na região sul, dois no Centro-Oeste e dois no Sudeste. Nesta ocasião, nenhum parâmetro relacionado às funções educacional e de supervisão foram relatados como concluídos, enquanto o parâmetro concluído com maior frequência foi o de equipe multiprofissional com atuação interdisciplinar (relacionado a função assistencial), concluído em 18% dos ambulatórios

A Tabela apresenta o consolidado de diagnóstico dos parâmetros nas duas etapas de avaliação. Na segunda autoavaliação, realizada em 2020, 93,7% (n = 15) dos ambulatórios referiu ter, pelo menos, um parâmetro “Concluído”. Aquele com maior percentual de aumento foi o de equipe multiprofissional, seguido pela utilização de mesmos critérios pelas equipes da APS e AAE para estratificação de risco dos usuários, ambos relacionados à função assistencial. Ainda no contexto da função assistencial, o parâmetro da atenção contínua foi o que apresentou menor percentual de conclusão no segundo momento. Cabe destacar que este foi o único parâmetro assistencial não implantado em nenhum dos ambulatórios na primeira etapa de avaliação.

Observa-se que o parâmetro com menor diferença de conclusão entre as etapas deste estudo foi o de organização de eventos voltados à educação de usuários em parceria com as equipes de APS do território, relacionado à função educacional. Entre aqueles relacionados à função educacional, os de encontros entre as equipes da APS e AAE para desenvolvimento da gestão de caso, e os de promoção de capacitação integrada para profissionais da APS e AAE foram os que apresentaram maior percentual de conclusão em 2020.

No contexto da função de supervisão, destaca-se maior aumento do relato de conclusão do parâmetro de vínculo estreito de comunicação entre profissionais da AAE e APS.

A Figura apresenta a diferença da soma dos parâmetros concluídos em cada uma das três funções da AAE estudadas nas duas etapas de autoavaliação, de acordo com a localização das unidades nas regiões geográficas brasileiras.

## DISCUSSÃO

O estudo descreveu parâmetros relacionados a três funções do modelo Pasa em ambulatórios distribuídos nas cinco regiões geográficas brasileiras. Evidenciou-se que, antes da execução da metodologia da PAS, por meio do projeto PlanificaSUS, a função assistencial pareceu estar parcialmente implantada nos ambulatórios do Sul, Sudeste e Centro-Oeste, no entanto, as funções educacional e de supervisão não eram desenvolvidas em nenhum dos ambulatórios estudados. No segundo momento de avaliação, identificou-se avanços importantes nas três funções previstas no modelo Pasa, em especial da função assistencial. Acredita-se que tais avanços contribuíram de forma relevante para a organização dos processos de trabalho que potencializam o acesso e cuidado de gestantes e crianças de alto risco nas diferentes regiões geográficas brasileiras.

Na primeira etapa de avaliação do estudo, somente os parâmetros relacionados à função assistencial apareceram como processos já implantados, o que corrobora com a premissa que o modelo em silos vigente trabalha somente com essa função^6^. No entanto, apesar de já estar parcialmente implantada, a conformidade dos seus parâmetros foi verificada na minoria dos ambulatórios.

O parâmetro assistencial que mais foi desenvolvido entre as duas etapas de autoavaliação refere-se à equipe multiprofissional com atuação interprofissional. Peduzzi^15^ destaca que “o trabalho em equipe multiprofissional consiste no trabalho coletivo que se configura na relação recíproca entre as múltiplas intervenções técnicas e interação dos agentes de diferentes áreas profissionais”. Neste sentido, para a equipe não se configurar em “equipe agrupamento”, é fundamental que ocorra comunicação e articulação das ações entre os atores, na perspectiva da integração do trabalho, considerando um projeto assistencial comum, flexibilidade da divisão de trabalho e autonomia técnica interdependente, a partir das diferenças nas especialidades^16^. Desenvolver competências para o trabalho em equipe, refletir e discutir criticamente a atuação dos profissionais, bem como a definição dos limites e autonomia profissional é fundamental para romper com o modelo de formação e as práticas em saúde fragmentadas, visando fortalecimento do trabalho multiprofissional e interdisciplinar para responder às necessidades de saúde da população^17^.

Destaca-se que estes aspectos corroboram e são oportunizados pelo modelo Pasa, o qual prevê atividades assistenciais desenvolvidas na forma de atenção contínua, a partir de uma diretriz clínica por uma equipe multiprofissional com atuação interprofissional^6,13^. Tais características representam uma diferença marcante do modelo Pasa em relação ao modelo em silos. Na perspectiva do ciclo de atenção contínua, preconiza-se uma avaliação integrada e a construção coletiva do plano de cuidados, a partir de atendimentos individuais sequenciais, em ordem previamente estabelecida^18^. Este estudo identificou que nenhum ambulatório ofertava atenção contínua na primeira etapa de avaliação, enquanto na segunda etapa, após cerca de um ano de execução da metodologia da PAS por meio do PlanificaSUS, cerca de 20% já desenvolviam atividades assistenciais na forma de atenção contínua. É importante destacar que se previa o início dos processos para a implantação da atenção contínua nos ambulatórios participantes do PlanificaSUS durante o primeiro trimestre de 2020. No entanto, esta ocasião coincidiu com o marco de início da pandemia de covid-19 no Brasil, sendo necessária a adaptação das atividades para modalidade virtual e o replanejamento de cronogramas, devido à necessidade de priorização do atendimento às demandas da covid-19. Neste sentido, tal avanço é considerado relevante, apesar de ser o parâmetro da função assistencial que menos foi desenvolvido no período estudado.

Foram verificados também avanços de outros parâmetros relacionados à função assistencial, os quais remetem à integração entre equipes da atenção primária e do ambulatório, pois abordam questões relacionadas ao alinhamento de diretrizes clínicas entre os serviços, regulação do acesso, fluxos e compartilhamento do cuidado. Com a qualificação dos processos de trabalho, por meio da metodologia da PAS, os ambulatórios aprimoraram a integração com a APS. Em especial no que se refere à atenção materno-infantil, cabe destacar que a Rede Cegonha preconiza o acesso das gestantes de alto risco aos ambulatórios especializados em tempo oportuno^7,10^. Para que isso ocorra, é necessário que haja integração em rede e uma boa comunicação entre os profissionais de saúde. Nesta perspectiva, para que o compartilhamento do cuidado entre serviços de APS e AAE seja assertivo, deve-se considerar a construção de vínculos entre usuários e profissionais, mas também entre profissionais de diferentes serviços, com o reconhecimento do papel da APS como coordenadora do cuidado^6,7,13,18^.

No que se refere à função educacional do modelo Pasa, este estudo evidenciou uma lacuna acerca da organização de eventos voltados à educação de usuários pelos ambulatórios em parceria com a APS. Apesar disso, cabe reforçar que o modelo Pasa preconiza o desenvolvimento de ações educacionais durante o ciclo de atenção contínua, voltadas aos usuários, para fortalecer a integralidade, a educação em saúde, o autocuidado e o autocuidado apoiado^6,13,18,21,22^.

No que se refere à função educacional dos ambulatórios com as equipes da APS, os resultados deste estudo evidenciam a sinergia do modelo Pasa com a educação permanente em saúde^6,13,17,18,23^. Ainda que de forma pouco expressiva, identificou-se um aumento da implantação de atividades em conjunto com a APS, apontando para o fortalecimento do apoio matricial desenvolvido entre equipes, em que os profissionais dos ambulatórios asseguram a retaguarda técnico-pedagógica e técnico-assistencial aos profissionais de referência^6,13,18,23^. Tais estratégias valorizam a aprendizagem significativa no trabalho, por meio das trocas entre especialistas dos ambulatórios e equipes de referência da APS, com grande potencial de transformação de práticas no cotidiano dos serviços^24,25^.

O estudo evidenciou pequenos avanços no que se refere ao vínculo estreito de comunicação entre profissionais dos ambulatórios e da APS e às ações de supervisão, com o objetivo de apoiar essas equipes na melhoria dos processos relacionados à linha de cuidado materno-infantil. Destaca-se que a supervisão/ apoio institucional contribui para uma gestão compartilhada e participativa entre serviços, gestores, profissionais e usuários. Esse apoio promove desenvolvimento institucional, uma vez que pressupõe mediação entre saberes técnicos e compromisso ético-político, considerando que toda gestão é produto das relações entre pessoas^26^. Tal referencial corrobora a práxis da função de supervisão de ambulatórios organizados pelo modelo Pasa, o qual busca romper com as lógicas tradicionais e burocratizadas de comunicação entre diferentes pontos da RAS.

Diversos fatores podem estar relacionados às mudanças evidenciadas por este estudo nos processos de trabalho de serviços especializados de acordo com o modelo Pasa. Uma revisão sistemática apresenta facilitadores e barreiras para a implementação de inovações na atenção secundária^29^. Entre as principais barreiras, destacaram-se a falta de recursos humanos, materiais e financeiros, de integração do fluxo de trabalho e de prontidão organizacional. Por outro lado, uma cultura organizacional aberta e de apoio, formação, educação e conhecimento, bem como o reconhecimento do valor agregado aos usuários representaram facilitadores para a implementação de inovações em serviços especializados^29^.

Nesse contexto, a metodologia da PAS prevê elementos para mitigar algumas destas barreiras e facilitar o processo de implementação de serviços ambulatoriais no modelo Pasa. Entre estes, destacam-se a pactuação de diretrizes clínicas, fluxos e protocolos na rede de atenção, momentos de alinhamento teórico e conceitual, assim como de reflexão, discussão e pactuações da prática de trabalhos, por meio do processo de tutoria.

Ainda que prevaleçam os desafios do acesso à atenção especializada, avanços têm ocorrido em termos de reconhecimento e papel da APS como ordenadora da RAS^30,31^. Destaca-se ainda a recente publicação da Política Nacional de Atenção Especializada em Saúde^32^, a qual preconiza diretrizes, dimensões e eixos estruturantes que visam a ampliação do acesso, o cuidado centrado nas necessidades das pessoas, maior integração entre as práticas profissionais, estímulo à educação permanente e o fortalecimento da APS resolutiva, ordenadora e coordenadora do cuidado.

Entre as limitações deste estudo, destaca-se o viés de informação que pode ter ocorrido, uma vez que foram considerados dados secundários provenientes de autoavaliação de representantes dos serviços de saúde. Entre as estratégias realizadas para mitigar a possibilidade de divergência do relatado pelos profissionais e do que realmente existe nos ambulatórios, cabe pontuar o uso de instrumento padronizado e o apoio de atores externos durante as atividades de autoavaliação pelos serviços previstas no PlanificaSUS. Entre os pontos fortes, destaca-se que o estudo foi o primeiro a descrever a organização de ambulatórios conforme previsto pelo modelo Pasa, a partir da metodologia da PAS, incluindo unidades de todas as regiões geográficas brasileiras.

Além disso, destaca elementos que dialogam com a estruturação e organização da atenção especializada no modelo Pasa, na perspectiva das RAS. Particularmente, a relevância da metodologia da PAS no que tange ao fomento de reflexões e indução de práticas que visam à (re)organização do processo de trabalho das equipes de saúde na qualificação do acesso e cuidado de gestantes e crianças de alto risco na RAS.

Evidencia-se a necessidade de futuras pesquisas de implementação, com vistas a identificar os facilitadores e barreiras para a organização de ambulatórios de forma integrada com a APS nos diferentes contextos brasileiros. Em especial, estudos que contemplem a organização da atenção especializada no modelo Pasa são necessários não apenas para investigar os determinantes da implementação, mas também seus desfechos, que se referem às mudanças e à sustentabilidade das práticas dos profissionais de saúde e gestores de forma multiprofissional, colaborativa, baseada em diretriz clínica, integral e equânime e ao impacto no cuidado de gestantes e crianças de alto risco.

## CONCLUSÃO

Este estudo possibilitou verificar o avanço na implantação das funções assistencial, educacional e de supervisão, conforme o modelo Pasa, em ambulatórios que adotaram a linha de cuidado materno-infantil como prioritária no PlanificaSUS. Além disso, ao identificar apenas parâmetros da função assistencial na primeira etapa de avaliação, ainda que incipientes, evidencia-se a predominância do modelo em silos, fragmentado no cenário brasileiro. A metodologia da PAS contribuiu com a (re)organização do processo de trabalho das equipes de saúde e induziu mudanças de práticas de cuidado à saúde materno-infantil em ambulatórios de forma integrada com a APS, de acordo com o modelo Pasa. A partir desta pesquisa, desdobram-se necessidades de outros estudos para elucidar o impacto da organização de ambulatórios integrados em rede com a APS nos desfechos em saúde da população brasileira.
